# The Hasson Versus Veress Trocar Wars: Determining the Safety Index of Laparoscopic Surgical Entry Techniques

**DOI:** 10.7759/cureus.74073

**Published:** 2024-11-20

**Authors:** Izna Najam Syed, Noem Najam Syed, Rabail Naseem, Deepak Singh-Ranger

**Affiliations:** 1 General Surgery, The Royal Wolverhampton NHS Trust, Wolverhampton, GBR; 2 General Surgery, Dr. Ruth K. M. Pfau Civil Hospital Karachi, Karachi, PAK; 3 Medicine, Kettering General Hospital, Kettering, GBR; 4 Colorectal Surgery, The Royal Wolverhampton NHS Trust, Wolverhampton, GBR

**Keywords:** hasson's technique, laparoscopic surgery, primary port placement injuries, safety index, veress needle

## Abstract

Background: Classically, there are two techniques for establishing pneumoperitoneum in laparoscopic and robotic surgeries: the closed Veress needle technique and Hasson's technique for open placement of laparoscopic ports. Most surgeons prefer the open Hasson technique, even though it is not the gold standard. Some surgeons still favour the Veress needle technique despite literature linking it to visceral and vascular damage.

Aims: This study aimed at determining the safety index of the two techniques of establishing pneumoperitoneum in laparoscopic and robotic surgeries by evaluating the risk of injuries associated with Veress and Hasson's techniques.

Materials and methods: This is a retrospective cohort study evaluating the incidence of primary port placement injuries (PPPI) using Hasson's open trocar technique and Veress needle technique in 200 laparoscopic and robotic cases (emergency vs. elective) over a three-month period (January to March 2024) at Newcross Hospital. The exclusion criteria were secondary port injuries. IBM SPSS Statistics for Windows, Version 24 (Released 2016; IBM Corp., Armonk, New York) and Microsoft Excel (Microsoft Corporation, Redmond, Washington) were used for data analysis.

Results: Hasson's open technique of primary port placement was used in 74% of emergency cases, while the Veress needle technique was used in 26% of emergency surgeries. Similarly, the most common technique for establishing pneumoperitoneum employed in elective surgical procedures was Hasson's open technique (77%). When comparing the safety index of the two techniques for establishing pneumoperitoneum, we found that there were no major PPPI associated with either technique, with minimal incidence of minor PPPI associated with both techniques. While some cases did involve injuries from secondary port insertions (bowel and vascular injuries), these were excluded according to our exclusion criteria. Additionally, no perioperative mortality associated with primary trocar placement was observed.

Conclusion: Although the literature describes the association of the Veress technique with visceral and vascular injuries, our study found it to be as safe as Hasson's open port placement technique. Therefore, either technique can be employed for the safe establishment of pneumoperitoneum in laparoscopic and robotic surgeries.

## Introduction

In 1987, Philippe Mouret performed the first laparoscopic cholecystectomy. This breakthrough made laparoscopic surgery the preferred operative method for treating a vast array of abdominal pathologies, such as appendicitis and cholecystitis, as well as various gynaecological disorders [1]. Laparoscopic surgery is considered one of the most significant developments in the field of surgery. Further advancements in the form of the incorporation of robotic and digital technology have been revolutionary for surgical practice. Compared to conventional open surgeries, laparoscopy has significantly shortened patient recovery times and significantly improved the overall surgical quality [2,3]. Laparoscopic surgery has several benefits, including quicker recovery times, shorter hospital stays, less pain and discomfort after surgery, less disability, and better cosmetic outcomes (with less scarring), enabling patients to return to their daily life activities more quickly [4,5]. Despite the multiple advantages of laparoscopic surgery, the relatively blind nature of its initial entry technique remains a major concern for surgeons [6].

A pneumoperitoneum is crucial for laparoscopic surgery because it provides the necessary visibility and space for manoeuvring at the surgical site [7]. The creation of a pneumoperitoneum and the initial trocar insertion are critical steps in laparoscopic surgery. Approximately 50% of the complications that arise during these procedures are linked to access techniques and insufflation of the peritoneal cavity [8]. Existing data suggest that two out of every 10,000 laparoscopic procedures may result in vascular injury, and 3.3 out of 10,000 procedures may have more insidious complications that are associated with perioperative mortality. These statistics highlight the importance of a safe laparoscopic entry technique to reduce the associated morbidity and mortality, particularly in view of the rising number of re-entry during laparoscopic procedures. Over the last three decades, the rapid developments in the field of laparoscopic surgery have woven them into the core of general surgery. However, there is still disagreement over the best way to enter the peritoneal cavity [9-12]. The Hasson open approach and the closed Veress needle insertion (VNI) procedures are two frequently used laparoscopic entrance techniques [13]. In addition to their proponents and opponents, each technique has its own associated sets of risks and benefits, which has led to an ongoing discussion, dubbed the "Trocar Wars", about which approach is safer and more effective.

Despite relatively slow insufflation rates and potentially fatal complications from iatrogenic vascular and visceral injuries, VNI remains a widely used method of primary port placement (PPP) today [14]. The Veress needle technique entails a blind puncture of the anterior abdominal wall, followed by insufflation with CO_2_ [15]. Contrarily, Hasson's approach, first performed and introduced in 1970, involves making incisions through the layers of the anterior abdominal wall at a site close to the umbilicus (supraumbilical or infraumbilical), followed by insertion of the primary trocar under direct vision before creating a pneumoperitoneum by insufflating the peritoneum with CO_2_ [16]. Despite its ease of use and relatively speedy peritoneal access, the blind nature of the VNI technique makes the patients more susceptible to iatrogenic injuries [14]. However, as per existing literature, iatrogenic vascular and visceral injuries are associated with both Hasson's open tracer placement technique and the VNI technique, and the best approach is still up for debate over 30 years later. Interestingly, newer generations of general surgeons seem to prefer open surgery, influenced by a plethora of questionable data [9-12].

Various studies have reported variable rates of morbidity and mortality associated with each of the laparoscopic entry techniques. While open laparoscopy was reported in certain studies to have a mortality rate of zero, closed laparoscopy showed a mortality rate of 0.03% [17]. Moreover, some studies reported no statistically significant distinction between the mortality associated with the two methods. Often, open Hasson's technique is recommended as a safer substitute for the Veress needle approach when it comes to preventing iatrogenic injuries during abdominal access during laparoscopic and robotic surgeries [18]. However, some literature suggests that the challenges associated with both PPP techniques remain comparable, including potential vascular and visceral injuries [19]. Hence, the choice of the PPP technique should be carefully considered in view of the patient and procedural factors.

As laparoscopy becomes the gold standard mode of operation across various surgical fields, it is becoming more and more crucial to identify and address any potential complications associated with primary trocar placement and establishing pneumoperitoneum. For laparoscopic surgeries to have an optimal outcome, it is imperative to have maximal visibility, which in turn reduces the risk of iatrogenic injury to surrounding visceral and vascular structures. Research on this topic is ongoing, with the aim of providing surgeons with guidelines regarding the ideal method of peritoneal entry technique during laparoscopic surgeries, which will consequently improve postoperative patient outcomes and reduce the potential risks and complications associated with laparoscopic surgical procedures. This research is crucial in order to advance safe surgical practices.

This study aims to address the debate on the safety index of the two predominantly used primary trocar placement techniques: Hasson's open trocar technique and the closed VNI technique. This article was previously presented as a poster abstract at the 2024 ALSGBI Annual Scientific Meeting on the 5th and 6th of November, 2024.

## Materials and methods

Study design, duration, and population

This retrospective cohort study was conducted over a three-month period, from January 2024 to March 2024, at New Cross Hospital in Wolverhampton, United Kingdom.

Inclusion criteria

Our study included non-randomised patients between 17 and 95 years of age who underwent laparoscopic, laparoscopic-assisted, or robotic surgeries for general surgical, urological, obstetric, and gynaecological pathologies. The study sample included a total of 200 patients, with 100 emergency and 100 elective procedures. The choice of PPP technique was made by the primary surgeon performing the procedure.

Exclusion criteria

Any iatrogenic injuries associated with any of the secondary port placements were excluded from the study to focus solely on complications arising from primary port access during laparoscopic procedures.

VNI technique

**Figure 1:**
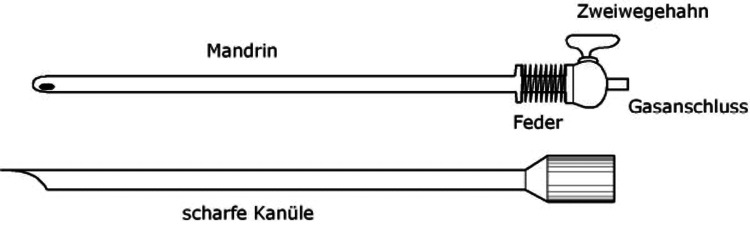
Veress needle. Attribution: Lx Zander via Wikimedia. This image is published under a Creative Commons license (CC BY-SA 3.0, https://commons.wikimedia.org/w/index.php?curid=4481680).

**Figure 2:**
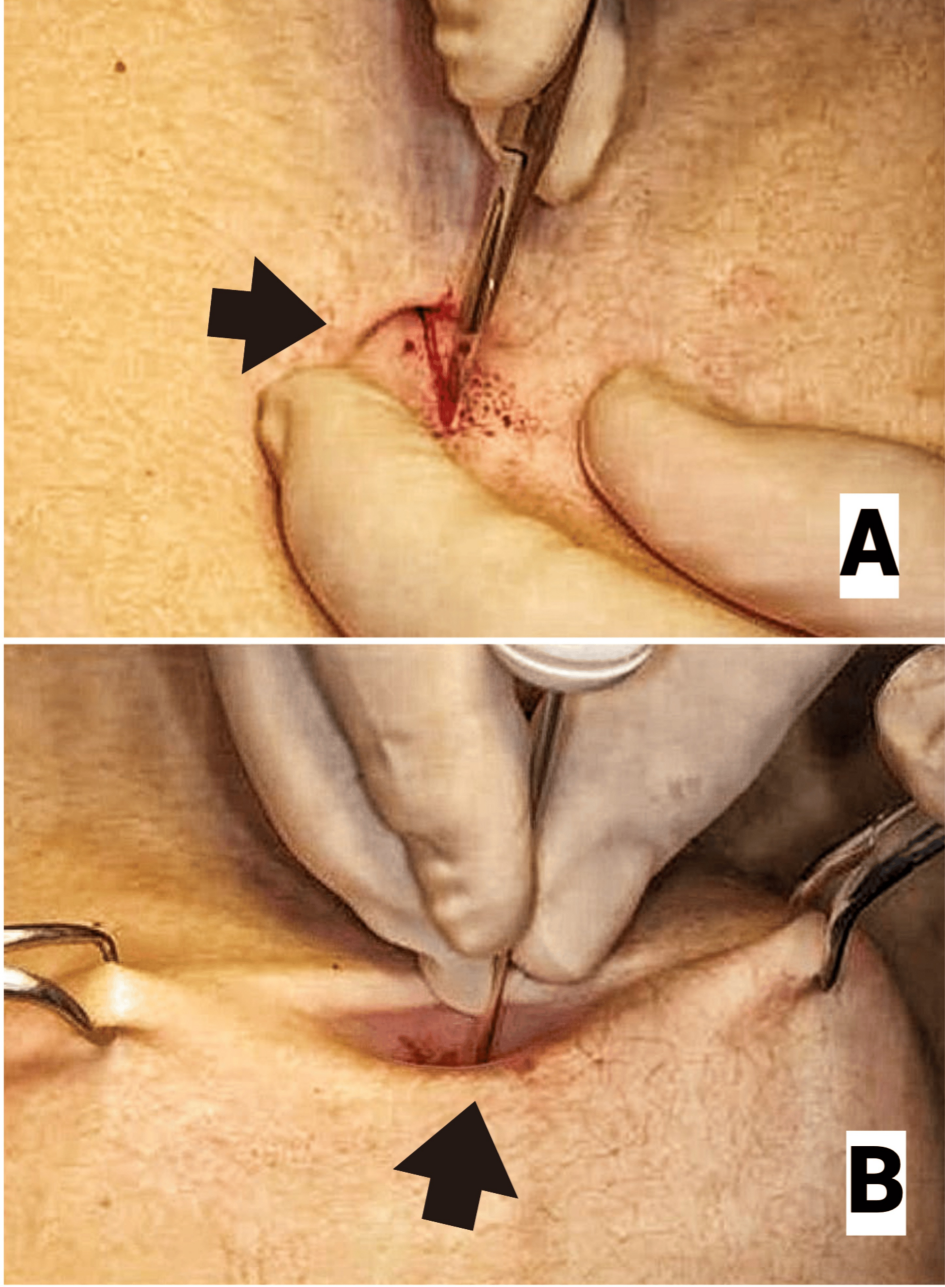
Technique for Veress needle insertion. Panel A shows the appropriate infraumbilical site of incision for the Veress needle. Panel B shows how the Veress needle is inserted safely through the incision site. Attribution: Alam et al. [20]. Permission was granted by Fahreyar Alam, the first author of the article. This image is published under the Creative Commons License.

When VNI (Figure 1) was used to access the peritoneal cavity, a 10 mm curvilinear incision was made either infraumbilically (Figure 2A) or at the Palmer's point (3 cm below the left costal margin in the mid-clavicular line), where infraumbilical access was contraindicated. To enable a safe and simple insertion of the Veress needle, the nondominant hand of the surgeon or the assistant elevated the abdomen wall in each case (Figure 2B). Two tests were used to confirm the entry into the abdominal cavity: the drop test and the double click sound.

Hasson's technique

**Figure 3:**
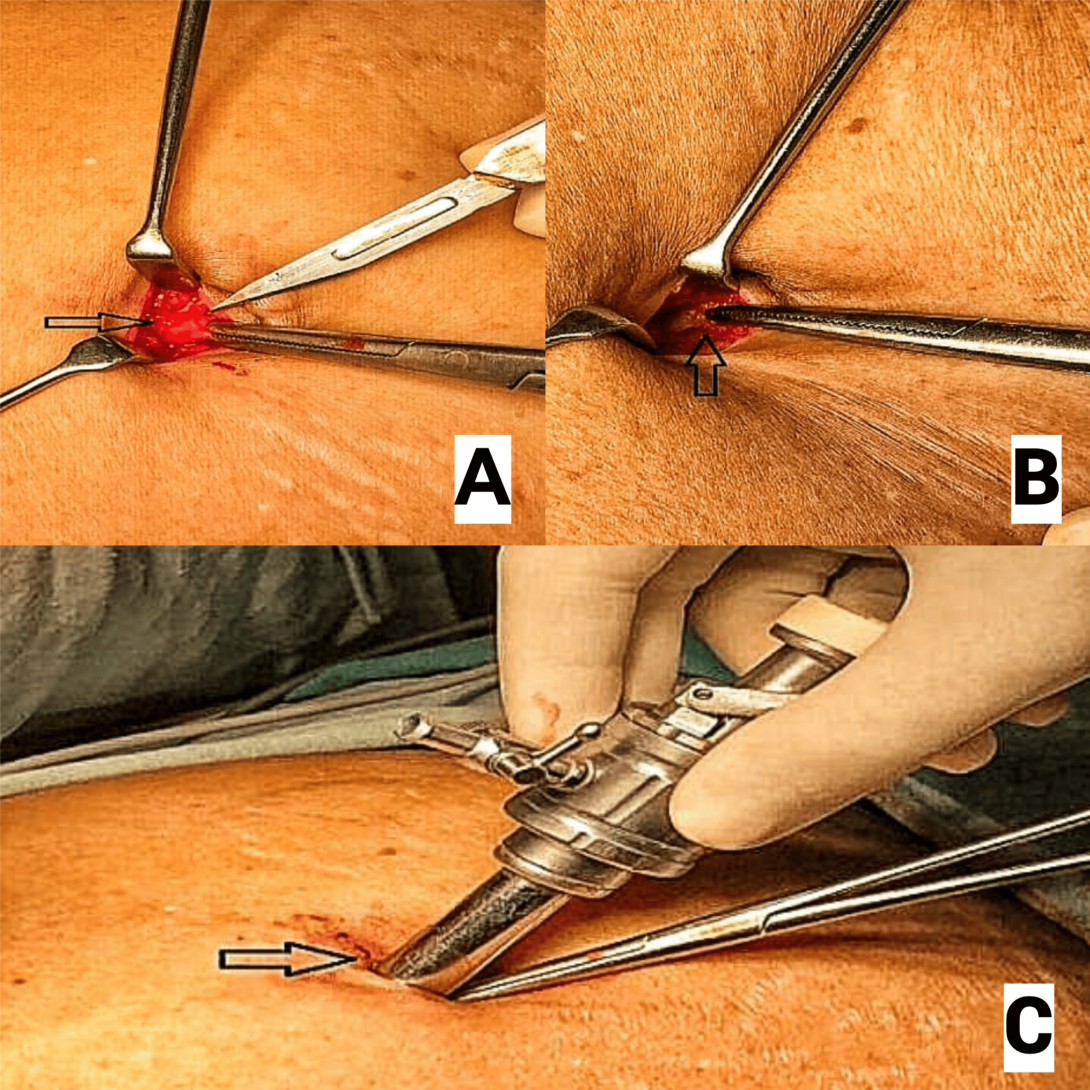
Hasson's technique. Panel A shows the rectus sheath through a small supraumbilical incision. Panel B represents the dissection of the rectus sheath. Panel C displays the insertion of a Hasson’s port. Attribution: Radhakrishna et al. [21]. Permission was granted by Veerabhadra Radhakrishna, the second author of the article. This image is published under a Creative Commons license.

**Figure 4:**
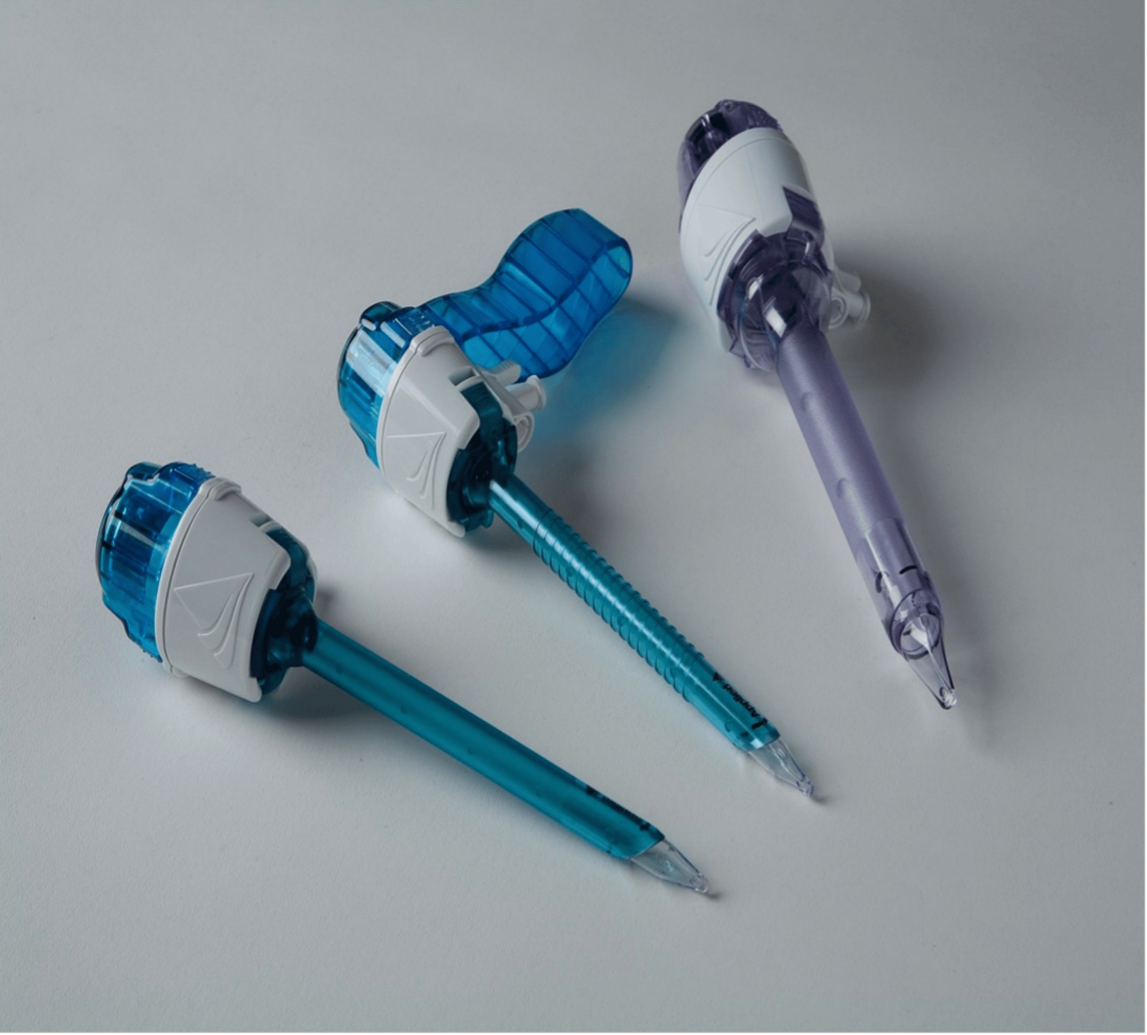
Disposable trocars used in Hasson's port placement technique. Attribution: Magnus1313 at English Wikipedia. This image is published under a Creative Commons license (CC BY-SA 3.0, https://commons.wikimedia.org/wiki/File:Disposable_Trocars.jpg).

When using Hasson's technique for PPP, a 10-12 mm semicircular or vertical infraumbilical or supraumbilical incision was made. The subcutaneous fat and tissue were bluntly dissected using artery forceps until the linea alba of the rectus sheath was visualised (Figure 3A). Once visualised, the fascia on either side of the linea alba was grasped using artery forceps, and a vertical 10 mm incision was made through the fascia (Figure 3B). Further blunt dissection was carried out to reveal the peritoneum, which was elevated using artery forceps and carefully opened using a scalpel. Once opened, the surgeon's index finger was introduced in the opening to confirm that the intraperitoneal space had been entered freely and to break down any omental adhesions. Subsequently, a 10-12 mm blunt-tipped Hasson's port (Figure 4) was introduced into the abdomen (Figure 3C).

Primary outcomes

This study evaluated the incidence of primary port placement injuries (PPPI) in 200 cases of laparoscopic, laparoscopic-assisted, and robotic surgeries using either the Hasson's open trocar placement technique or the Veress needle technique. These included complications such as vascular and bowel injuries, gas leakages, port site hematoma, port site infection, and umbilical hernia. Another variable assessed was perioperative mortality associated with both primary port insertion techniques.

Secondary outcomes

The secondary outcomes evaluated were the need for conversion to open surgery with both the Veress needle technique and Hasson's open technique.

Data analysis

The chi-square test was used for inferential analysis to investigate the relationship between the laparoscopic entry procedures (Hasson vs. Veress), the mode of surgeries, the intraoperative and postoperative complications, and conversion rates to open surgery. To guarantee accurate results in situations with small sample sizes, Fisher's exact test was utilised instead of the chi-square test when the predicted frequency in any cell was less than five (such as the relationship of the trocar placement technique with the associated complications). Each statistical test had a two-tailed design with a significance criterion set at p < 0.05. For statistical studies, Microsoft Excel (Microsoft Corporation, Redmond, Washington) and IBM SPSS Statistics for Windows, Version 24 (Released 2016; IBM Corp., Armonk, New York) were utilised. When appropriate, the inferential statistics' findings enhanced our comprehension of the data and enabled us to draw more defensible inferences on the relationship between surgical outcomes and the safety and efficacy of entry techniques.

## Results

Our study included patients aged 17 to 95 years, with a mean age of 46.47. We did not note any significant baseline differences in the two groups, age and gender (Table 1).

**Table 1:** Difference in baseline characteristics between the Hasson and Veress needle groups

Demographic Data	Hasson's Technique	Veress Needle Technique
Age (mean)	42	46
Gender (male:female)	92:59	20:29

Of the 100 emergency cases, the majority fell under general surgery (54%). Similarly, elective cases showed a higher general surgical distribution (65%) (Table 2).

**Table 2:** Distribution of emergency and elective laparoscopic cases by surgical specialties

Mode of Surgery	General Surgery	Gynaecology	Urology
Emergency cases (n = 100)	54	46	0
Elective cases (n = 100)	65	22	13

Additional details regarding the types of surgical procedures are presented in Tables 3, 4.

**Table 3:** Emergency laparoscopic surgical procedures

Surgical Procedure	Specialty	n
Laparoscopic appendicectomy	General surgery	43
Laparoscopic cholecystectomy	General surgery	11
Laparoscopic salpingectomy	Gynaecology	39
Diagnostic laparoscopy	Gynaecology	6
Laparoscopic removal of Mirena coil	Gynaecology	1

**Table 4:** Elective laparoscopic surgical procedures APER: abdomino-perineal excision of rectum

Surgical Procedure	Specialty	n
Laparoscopic cholecystectomy	General surgery	19
Laparoscopic anterior resection	General surgery	16
Laparoscopic APER	General surgery	9
Laparoscopic hemicolectomy	General surgery	10
Laparoscopic defunctioning colostomy	General surgery	3
Robotic anterior resection	General surgery	3
Robotic proctectomy	General surgery	5
Laparoscopic hysterectomy + bilateral salpingo-oophorectomy	Gynaecology	9
Laparoscopic bilateral salpingo-oophorectomy	Gynaecology	11
Laparoscopic tubal ligation	Gynaecology	2
Robotic radical prostatectomy	Urology	9
Robotic radical/partial nephrectomy	Urology	4

In emergency cases, Hasson's technique was employed for port insertion in 74% of instances, whereas the Veress needle technique was used to establish pneumoperitoneum in 26% of surgeries. In elective procedures, the open Hasson technique was the most used method for establishing pneumoperitoneum, accounting for 77% of the cases (Table 5).

**Table 5:** Proportion of Hasson's and Veress needle techniques in emergency and elective cases Test applied: chi-squared test

Mode of Surgery (Port Placement Technique)	Elective Surgeries (n = 100)	Emergency Surgeries (n = 100)	P-value
Hasson's open technique	77 (77%)	74 (74%)	0.62
Veress needle insertion	23 (23%)	26 (26%)	

A comparison of the safety index between the two techniques for establishing pneumoperitoneum revealed that neither group experienced any major injuries related to primary trocar insertion. One case of VNI was associated with a small haematoma formation at the primary port site. Although some cases did report secondary port insertion injuries affecting bowel and vascular structures, these were excluded from our analysis in accordance with our established exclusion criteria. Importantly, there were no observed instances of perioperative mortality associated with primary trocar insertion (Table 6).

**Table 6:** Primary port placement injuries (PPPI) associated with Veress needle insertion and Hasson's open trocar technique Tests applied: chi-squared test and Fisher's exact test

Complications	Veress Needle Insertion	Hasson's Open Technique	P-values
Gas leakage	0	3	0.38
Bowel injury	0	0	1.00
Vascular injury	0	0	1.00
Port site haematoma	1	0	0.078
Port site infection	0	0	1.00
Port site hernia	0	0	1.00
Perioperative mortality	0	0	1.00

There was no significant difference in frequencies of conversion to open surgery in either of the groups (Table 7).

**Table 7:** Comparison of conversion to open surgery in Veress needle insertion and Hasson's open trocar technique Test applied: chi-squared test

Conversion To Open Surgery	Veress Needle Insertion Technique	Hasson’s Open Trocar Placement Technique	P-Value
Yes	0	1	0.326
No	49	150	

## Discussion

Significant advancements have established laparoscopic surgery as a widely recognised and effective surgical technique across various surgical domains. The creation of pneumoperitoneum by CO_2_ insufflation is a crucial first step in laparoscopic surgeries, and several different techniques can be utilised to achieve this step. Establishing pneumoperitoneum is essential because it allows for a clearer surgical view and more space to operate; however, it also carries inherent risks, including iatrogenic injury to major blood vessels, such as the vena cava and iliac vessels, as well as potential harm to abdominal organs, including the intestine, liver, spleen, and omentum, during entry into the abdomen [22,23]. The Veress needle and open-entry methods are the two most commonly used techniques for establishing pneumoperitoneum. Despite their prevalence, there is still ongoing debate in the surgical community about which technique is superior in terms of safety and efficacy.

In our study, we included 200 patients aged 17 to 95 years, with the majority, 112 patients, being female. We found that neither the group with the VNI technique nor the one that underwent Hasson's open trocar placement technique reported any major bowel or vascular injuries. Our results were similar to other studies. Opilka et al. found the open method (Hasson) to be safer in their trials (54.84% safety rate), whereas the closed approach (Veress needle) had a safety rate of just 9.68%. Out of 5,347 procedures, 474 used the closed method, which reported three serious vascular injuries [18].

Comparing closed and open-entry approaches, Jansen et al. found that the complication rates were 0.7% for the closed VNI technique and 0.17% for Hasson's open techniques. They concluded that both techniques are safe [24]. Similarly, Chapron et al. [14] described non-randomised research that evaluated open and closed laparoscopic access techniques carried out by university-affiliated hospital teams. They found that the rate of bowel injury was 0.04% and major vessel injury was 0.01% with the closed technique, while the open technique had rates of 0.19% and 0%, respectively. In 2012, Bozkurt [25] in Turkey conducted a prospective study comparing the efficacy, complications, and postoperative pain between direct trocar access and open-entry methods. They reported that both the laparoscopic entry techniques have their pros and cons. They suggested that surgeons should use the laparoscopic entry technique that they are most familiar with and at ease with. In 2007 in Italy, Corcione et al. [26] noted that although all laparoscopic entry techniques carry some degree of risk, Hasson's technique is safer for patients with a history of previous surgeries.

Similarly, we found that only 1% of the patients who underwent VNI developed minor PPPIs, which included port site hematomas, port site infections, and port site hernias. In contrast, no minor PPPIs were observed in the group that underwent Hasson's trocar placement technique. As mentioned earlier, Jansen et al. [24] reported similar complication rates in the closed and open-entry techniques, leading them to conclude that both methods are safe and effective. Their conclusions align with the findings of our study. However, it is worth noting that Shakoor et al. [27] found a higher incidence of minor PPPIs associated with Hasson's technique compared to the VNI technique. This discrepancy highlights the variation in complication rates across existing literature and underscores the need for careful consideration when choosing a PPP technique in laparoscopic surgeries, as the selected method can impact the patient's postoperative outcome.

Furthermore, our study found that Hasson's open trocar placement technique is associated with a greater frequency of gas leakage, recorded at 1.5%. During laparoscopic surgeries, gas leakage is a significant issue, especially with open-entry techniques that frequently require specific equipment or additional suturing to avoid this problem. Open-entry procedures have been shown to have leakage rates of 15%, while closed Veress needle techniques have been shown to have leakage rates of 9.5% [28]. However, the rates of gas leakage with both PPP techniques were comparable, according to Virk [29]; this suggests that factors other than the entry method may influence leakage rates. Furthermore, Shakoor et al. [27] observed that compared to the open Hasson trocar placement technique, the VNI technique actually had a greater rate of gas leakage. These findings highlight the complexity of laparoscopic entry techniques and their varying impacts on procedural outcomes, underlining the need for cautious technique selection based on unique patient circumstances.

Our study also found no perioperative mortality associated with either laparoscopic entry technique employed. In 2021, Hassan [30] reached similar conclusions, reporting no instances of perioperative mortality with both the closed VNI technique and the open Hasson's port placement technique. Their findings corroborate the safety index of these laparoscopic entry methods. Similarly, a thorough analysis by Bonjer et al. [17] revealed no discernible variation in death rates between open and closed laparoscopic entrance procedures, with rates for open techniques recorded at 0% and for closed techniques at just 0.003%. These findings contribute to the growing body of literature suggesting that both approaches are safe and effective for entry into the abdominal cavity during laparoscopic procedures.

Moreover, this study found that the rates of conversion to open surgery due to the failure of establishing pneumoperitoneum after the closed VNI technique were minimal, with a rate of 0% for VNI and only 0.005% for the Hasson's open technique, indicating that pneumoperitoneum can be achieved with both procedures generally without requiring a conversion to open surgery. Conversely, Taye et al. [31] found that the group undergoing VNI had considerably greater failure rates in establishing pneumoperitoneum and, thus, the necessity for conversion to open surgery, with rates of 0.8% compared to 0.18% for open procedures. Furthermore, comparable conclusions were drawn by Ali et al. [32] and Akbar et al. [33], who reported failure rates for open versus closed approaches of 0% versus 8.57% and 0.72% versus 2.9%, respectively. These findings highlight the significance of selecting the appropriate peritoneal access technique in laparoscopic and robotic procedures, as variations in failure rates can have a huge impact on patient outcomes.

Limitations

The limitations of this study include its retrospective methodology, possible selection bias, and the brief three-month evaluation period. These may have impacted the study's generalisability. Furthermore, the generalisability is limited by the study being carried out at a single institution, and relying solely on pre-recorded data may result in the underreporting of minor injuries. Future studies with a larger sample size, multi-centre designs, and pre-designed proformas for recording minor and major PPPIs are needed to validate these findings and explore long-term outcomes associated with each technique.

## Conclusions

Although the existing literature often describes the potential association of the Veress needle technique with visceral and vascular injuries during laparoscopic procedures, our study found no statistically significant difference in the safety index between the Veress technique and the Hasson open port placement technique. This indicates that both methods are relatively safe when performed correctly. Consequently, it suggests that there is no universally optimal method for creating pneumoperitoneum in laparoscopic and robotic surgeries. However, randomised control trials with larger sample sizes are required to determine the safety index of both techniques with statistical certainty. Surgeons can choose either technique based on their experience, preference, and the specific clinical scenario they encounter. Ultimately, the decision should be guided by the individual surgeon's judgment and the unique circumstances of each case, as both techniques have their merits and can be effectively employed to achieve successful outcomes.
